# The Effects of Liking Norms and Descriptive Norms on Vegetable Consumption: A Randomized Experiment

**DOI:** 10.3389/fpsyg.2016.00442

**Published:** 2016-03-30

**Authors:** Jason M. Thomas, Jinyu Liu, Eric L. Robinson, Paul Aveyard, C. Peter Herman, Suzanne Higgs

**Affiliations:** ^1^School of Psychology, University of BirminghamBirmingham, UK; ^2^Department of Psychological Sciences, University of LiverpoolLiverpool, UK; ^3^Nuffield Department of Primary Care Health Sciences, Radcliffe Observatory Quarter, University of OxfordOxford, UK; ^4^Department of Psychology, University of TorontoToronto, ON, Canada

**Keywords:** social norms, liking, delay, maintenance, healthy eating, vegetables, low vegetable consumers, broccoli

## Abstract

There is evidence that social norm messages can be used to promote the selection of fruit and vegetables in low habitual consumers of these foods but it is unclear whether this effect is sustained over time. It is also unclear whether information about others' liking for a food (liking norm) could have the same effect. Using a 2 × 5 × 2 experimental design we investigated the effects of exposure to various messages on later intake from a food buffet and whether any effects were sustained 24 h after exposure in both low and high consumers of vegetables. There were three factors: delay (immediate food selection vs. food selection 24 h after exposure), message type (liking norm, descriptive norm, health message, vegetable variety condition, and neutral control message), and habitual consumption (low vs. high). The buffet consisted of three raw vegetables, three energy-dense foods, and two dips. For vegetables and non-vegetables there were no main effects of message type, nor any main effect of delay. There was a significant message × habitual vegetable consumption interaction for vegetable consumption; however, follow up tests did not yield any significant effects. Examining each food individually, there were no main effects of message type, nor any main effect of delay, for any of the foods; however, there was a message × habitual vegetable consumption interaction for broccoli. Consumption of broccoli in the health message and descriptive norm conditions did not differ from the control neutral condition. However, habitually low consumers of vegetables increased their consumption of broccoli in the vegetable variety and liking norm conditions relative to habitual low vegetable consumers in the neutral control condition (*p* < 0.05). Further, investigation of the effects of the liking norm and vegetable variety condition on vegetable intake is warranted. This trial is listed as NCT02618174 at clinicaltrials.gov.

## Introduction

It is well-established that a variety of social influences affect what and how we eat (Herman et al., 2003). For instance, when eating with a group of friends or acquaintances, we often eat more than if we had eaten alone (de Castro and Brewer, 1992; see Herman, 2015, for a review). However, when dining with another who eats either a small or large amount, we usually adjust our food consumption to match their intake (see Cruwys et al., 2015, for a review). It has also been reported that we may limit our intake when eating with people who we do not know to try to create a positive impression of ourselves (see Vartanian et al., 2015, for a review). This has been reported for both men and women (Pliner and Chaiken, 1990), although there is evidence that eating a small portion is viewed as feminine whereas eating a large portion is viewed as masculine; thus consumption also varies according to how individuals wish to present themselves (Pliner and Chaiken, 1990; see Vartanian et al., 2007, for a review). There is no question that social influences on eating are powerful and pervasive. We may ask, however, whether social influence can be used to promote healthy food choices.

Social norms reflect the behaviors and attitudes of individuals belonging to a group. For instance, in a group of students, descriptive social norms describe how students behave (e.g., “most students *eat* healthily”) while injunctive social norms reflect what students approve of (e.g., “most students *endorse* healthy eating”). Recent work by Robinson et al. (2014) examined the potential of social norm messages to enhance fruit and vegetable consumption in two laboratory-based studies using descriptive norms and injunctive norms. In their first experiment, they reported that after exposure to a descriptive social norm message suggesting that most students eat plenty of vegetables, participants ate more vegetables at a buffet lunch than when they were given information about the health benefits of eating vegetables. Similar results were reported in a second study for the descriptive norm message, but providing information that other students approve of the consumption of lots of fruit and vegetables (an injunctive norm) did not affect later intake. Furthermore, in both studies, the effect of the descriptive norm message was present only for those who were low habitual consumers of these foods. High consumers did not alter their intake in response to the social norm message, presumably because they were already behaving in line with the norm. The effectiveness of the descriptive norm over the injunctive norm in the study by Robinson et al. (2014) has been observed by others. Stok et al. (2014) reported that fruit intake by students was enhanced by a descriptive but not an injunctive norm message. Further, Lally et al. (2011) showed that descriptive norms, but not injunctive norms, are significant predictors of food consumption. Injunctive norms have less of an effect to change eating behavior than descriptive norms because people already approve of eating fruit and vegetables even if they do not eat many vegetables, hence there may be a limited capacity to further change this attitude. These results do hint that the type of norm message used in intervention studies is important, but to date few studies have investigated the effect of different types of norm messages on food selection and food intake.

It has been argued that social norms may influence eating behavior by altering expected liking for a food (Higgs, 2015). Supporting this idea, work by Robinson and Higgs (2012) has shown that participants' expected liking for orange juice was influenced by providing them with social normative information on how well orange juice was liked by other students (see Higgs, 2015 for the most recent discussion on liking). Hence, it is plausible that a norm suggesting that other students like eating vegetables might enhance expected liking of these foods, and by extension, enhance their selection and consumption, but this remains to be tested. It is also possible that a norm emphasizing liking for vegetables might be effective in increasing intake of vegetables that are not very well liked, for example, cruciferous vegetables that have a bitter taste. Typically, previous studies of the effects of social norms on eating have not included such vegetables.

A current limitation of using social norm messages to enhance healthy eating is that it is unclear whether social norm effects on food intake are sustained over time (Robinson, 2015). Much of the previous work has focussed on assessing food intake immediately after exposure to the norm (Robinson et al., 2013, 2014). There is some evidence that social norm effects on other health-related behaviors may persist beyond initial exposure. For instance, research using social norm messages to reduce alcohol consumption has shown that the effects are maintained at 1-, 3-, and 6-month follow-up (Neighbors et al., 2004; Lewis and Neighbors, 2007). In addition, the study by Stok et al. (2014) reporting increased fruit intake in response to a descriptive norm, was based on self-reported fruit intake over a 2 day period. These data suggest that the effect of providing norm information might persist beyond initial exposure but this possibility remains to be tested for effects on actual intake.

A more general point about social norms studies concerns which control conditions provide an appropriate comparison. Previous work has compared social norm effects to: health-based messages (Robinson et al., 2014); messages about exam preparation (Robinson et al., 2013); and conditions in which no information is provided (Mollen et al., 2013). No previous studies have compared the effect of a social norm message to a poster that refers to food in a neutral context, devoid of any social or health information, in order to determine whether simply exposing the concept of vegetables has an effect on later intake.

The aim of the present study was to test whether a liking norm about vegetable consumption would enhance the intake of vegetables by habitual low consumers of vegetables and whether the effects would be sustained over a 24-h delay. This norm conferred neither what people do, nor what they approved of (descriptive and injunctive norms), but instead conveyed what people liked (a liking norm). We hypothesized that relative to a neutral control condition that did not mention vegetables; the liking norm would significantly enhance the consumption of a range of vegetables (including a cruciferous vegetable) by low consumers. We further hypothesized that this effect might be sustained over a 24-h delay. We also examined three positive controls: the effects of a descriptive social norm message about vegetable consumption and a health-based message (as these have been used previously), and a condition that mentioned vegetable variety but did not mention normative consumption or the health benefits of consuming vegetables (to examine simple exposure effects to the mention of vegetables). Each of these were compared to the neutral control condition which did not mention vegetables, but was related to the age of the University of Birmingham. A *post-hoc* study was also conducted on the messages to investigate the potential mechanisms by which they exerted their effect.

## Methods and materials

### Participants

Four hundred participants consented to take part in the study. Participants were excluded from participating at the start of their test session if they were not a fluent English speaker. They were also excluded if they were a smoker or a diabetic, had any food allergies, or any past or present episodes of depression, anxiety, or an eating disorder (to avoid any effects of these factors on participant eating behavior). Therefore, 23 were excluded at the start of their test session based on these study criteria. A further 24 participants were excluded after completing the study as they were unable to recall the poster message correctly (responses were coded as correct or incorrect by a single coder). Thus, 353 successfully completed testing. The final group comprised 253 women and 100 men with a mean body mass index (BMI) of 22.6 kg/m^2^ (*SE* = 0.2) and a mean age of 21.5 years of age (*SE* = 0.2). Participants were recruited from the University of Birmingham via posters and from the School of Psychology Research Participation Scheme. To reduce the likelihood of participants guessing its aim, the study was advertised as two separate experiments conducted by different researchers: (1) An educational poster rating study; (2) A study examining appetite and mood. Participants were reimbursed with course credits or £15 cash for participating. Ethics approval was provided by the University of Birmingham Ethics and Research Governance Committee and informed consent was obtained from all participants. The study was conducted in accordance with the ethical standards laid down in the 1975 Declaration of Helsinki, as revised in 1983.

### Design

A between-subjects design was used that had three factors: Delay (no delay and delay), Message (neutral control, vegetable variety condition, health, descriptive norm, and liking norm), and habitual consumption (low vs. high). Participants in the No Delay condition saw the poster message and chose from the food buffet within the same test session. Participants in the Delay condition saw the poster message on the first day and then returned 24 h later to choose from the food buffet. The sample size was based on previous research that tested comparable numbers of participants (Robinson et al., 2014). Participants were randomly allocated to conditions using a randomization website (www.randomizer.org). Gender was specified as a variable to balance, hence the randomization resulted in a testing order that would ensure a balanced ratio of males:females in each condition. Test sessions took place from 10 a.m. to noon and 2 to 5 p.m. on week days.

### Questionnaires

A questionnaire that included questions on age, gender, medical illnesses, food intolerances, psychiatric issues, and smoking and drinking habits (used previously by Thomas et al., 2015) was used to gather demographic information and to exclude participants based on the study criteria. An eating questionnaire, which consisted of two open-ended questions asking participants what they had eaten and drank that day and when (based on a similar measure used by Thomas et al., 2014) was used to check that the participant had not eaten for 2 h prior to attending the laboratory. To maintain the cover story for the first study, participants completed a poster evaluation questionnaire, rating the poster on key aspects (trustworthiness, believability, relatability, meaning, clarity, comprehension, and professional appearance) using a five-point Likert scale with the response scale ranging from strongly disagree to strongly agree (based on a similar measure used by Robinson et al., 2014). The Three Factor Eating Questionnaire (TFEQ—Stunkard and Messick, 1985) was used to assess whether there were differences in eating styles between conditions. Visual Analog Scales (VAS) were used to assess mood and appetite. VAS items included: alert, drowsy, light-headed, anxious, happy, nauseous, sad, withdrawn, faint, hungry, full, desire to eat, and thirst. Participants indicated how much they felt a particular state, by marking on a 100 mm horizontal line, between the anchors “Not at all” and “Very.” Participants also completed a VAS scale similar to that described above to rate their liking for buffet foods. They were also asked to record using a tick box response whether they dipped the food in the dip provided. Usual vegetable intake was assessed using two open-ended questions asking “How many servings of vegetables do you normally eat a day?” and “Think back carefully—How many servings of vegetables did you eat yesterday?” (used by Robinson et al., 2014). Participants completed questions asking whether they thought that anything from the “first study” affected their behavior in the “seconds study” (in order to check whether they were aware that the studies were linked). They were also asked what percentage of students they thought met the recommended guidelines for fruit and vegetable intake (VAS scale with the anchors 0 and 100%) and what the usual intake of vegetables was for other students (scale from 1 to 10 servings per day). Finally, they were asked to recall the salient points from the poster that they had seen earlier using an open-ended response format.

### Food stimuli

A buffet consisting of six bowls of snack food items and two pots of dipping sauces was provided to participants: raw cucumber slices (160 g, 18 calories), raw celery sticks (140 g, 14 calories), raw broccoli florets (100 g, 33 calories), ready-salted Pringles (60 g, 313 calories), ready-salted tortillas chips (60 g, 293 calories), Ritz crackers (80 g, 404 calories), salsa dip (100 g, 29 calories), and a paprika yogurt dip (100 g, 49 calories). The dips were provided to facilitate the consumption of these foods (which are often served with dips in the UK). Food weights were selected so that bowls would appear to be full and visually matched in terms of the amount of food available. Each bowl of food was weighed before it was given to the participant and after they had finished eating to provide a measure of how much food was selected. Any selected food that was uneaten (taken from a bowl but not consumed) was subtracted from this, to yield an intake measure of food that was both selected and consumed.

### Messages

Posters were used to display the messages, with the message placed in the center surrounded by four supporting images (one at each corner). For the Neutral Control poster, images of the University of Birmingham were used; all other posters were surrounded by images of vegetables—courgette, peppers, carrots, and broccoli (identical images and placement for each poster). The text of the messages read as follows. Neutral Control: “Did you know that The University of Birmingham is over 100 years old? According to a recent survey, most students prefer to study at a University with an established record^*^.” Vegetable Variety condition: “Did you know there are more types of vegetables than you might realize? According to the latest estimate, there are over one thousand different types of vegetables^*^.” Health message: “Did you know eating a lot of vegetables is good for your health? Although, a lot of people aren't aware, heart health and cancer risk can be improved by eating over three servings of vegetables each day^*^.” Descriptive Norm message: “Did you know most students eat a lot more vegetables than you might realize? Although, a lot of people aren't aware, the typical student eats over three servings of vegetables each day^*^.” Liking Norm message: “Did you know more students like vegetables than you might realize? Although, a lot of people aren't aware, 80% of students actually like vegetables a lot^*^.” In each case, as the bottom of the poster, the following was inserted “^*^University of Birmingham Study.” All statistics for the messages were derived from data collected in self-reported pilot studies conducted with undergraduate students (data not shown).

### Procedure

**Figure 1:**
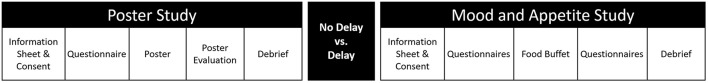
**Summary of Experimental procedure**.

An overview of key points during the procedure can be seen in Figure 1 below.

On arrival at the laboratory, the participant completed the “Poster Study.” They first completed a brief information sheet and consent form and then provided answers to the demographic questions, confirmed that English was their first language and answered questions to assess baseline perception of social norms (i.e., servings of vegetables a typical student eats each day). Participants were then handed the poster corresponding to their condition and asked to read it carefully twice. They then completed the poster rating questionnaire, after which they were provided with the debrief form for the “Poster study,” which suggested that we were interested in the ratings of a variety of educational posters. Participants in the No Delay condition were then taken to a different room with a different researcher to complete the “appetite and mood study.” Those in the Delay condition were told to attend the different room the following day at the same time. On arriving at the second testing venue, participants were presented with a new information sheet and consent form. They then completed the Lifestyle Questionnaire and Eating Questionnaire before completing a baseline set of VAS assessing mood and appetite. Then, they were provided with the snack food buffet on a trolley, were provided with a plate and a glass of water and instructed to select and consume whatever they wished. To corroborate the cover story for this “study,” when leaving, the researcher left a new set of VAS with the participant with instructions to complete it immediately after eating. Participants selected and consumed their food alone. After consuming the food and completing the VAS, the participants were asked to complete the food liking ratings and TFEQ. They were then asked to guess the aims of the study, report on their usual vegetable intake, and complete the manipulation checks. Weight and height were then measured using a stadiometer and digital scales. Participants were then asked if they had realized that we were interested in whether exposure to the message affected later food intake and were finally debriefed and compensated for their time.

### *Post-hoc* poster study

To better understand how the posters might have affected eating behavior we subsequently conducted a small study with 40 new participants (32 women, 8 men; mean age = 20.7, *SE* = 0.7). Participants were shown each of the posters from the study above (order randomized across participants) and asked to answer questions after viewing each one. Participants were first asked to report how they thought the poster might affect other people's eating behavior (open response). Two researchers coded the written responses, identifying the following five themes: (1) reference made to health (e.g., suggesting health as a reason to consume more health); (2) reference made to norms (e.g., noting the actions of others as an influence on food choice); (3) reference made to liking of vegetables (e.g., suggesting that the poster might affect how much vegetables are liked and hence how much they consume); (4) poster will increase variety of vegetables consumed; (5) poster will increase amount of vegetables consumed. Participants were also asked to report: the number of portions of vegetables they ate per day (0, 1, 2, 3, 4, 5, or more than 5); whether they thought the poster might influence the amount of vegetables eaten by other people (VAS Scale, 0–100 mm, Decrease Consumption-Increase Consumption); whether they thought viewing the poster might influence liking of vegetables by other people (VAS Scale, 0–100 mm, Decrease Liking-Increase Liking); what they thought the recommended number of portions of vegetables they should eat per day was (0, 1, 2, 3, 4, 5, or more than 5); What proportion of the population they thought consumes the recommended portions of vegetables (VAS Scale, 0–100 mm, 0% of the population–100% of the population); and finally, how many portions of vegetables they thought people eat per day based on the poster they read (0, 1, 2, 3, 4, 5, or more than 5).

### Analysis

#### General

One-way ANOVA was used to investigate main effects of message for baseline data (e.g., age), poster evaluations (e.g., poster understanding), etc., and independent *t*-tests were used to follow-up these effects. *T*-tests compared the neutral control message to all the other message types and the Bonferroni correction was applied.

#### VAS

To establish a factor structure for the appetite and mood rating scales, a principal components analysis (PCA) was run with varimax rotation. Analysis of the 13 items provided three factors with eigenvalues > 1, accounting for 57.3% of the variance. Items that loaded > 0.5 onto a factor were included, resulting in three factors of three or more items: appetite (hunger, fullness [reversed], desire to eat, and thirsty), negative mood (sad, withdrawn, happy [reversed], and anxious), and negative physical effects (lightheaded, nausea, and faint). Scores for each of the factors were calculated by summing the scores for all items in that factor, and then dividing by the number of items. Items with a negative scale were inverted to match the other items. Alertness and drowsiness did not significantly load onto a factor and were examined separately.

#### Poster ratings

The same PCA analysis described above was run on the seven poster items. Two factors emerged with eigenvalues > 1, accounting for 59.9% of the variance: legitimacy (trustworthiness, believability, and relatability of poster) and understanding (meaning, clarity, and comprehension of poster). Professional appearance did not significantly load onto either factor and so was examined separately.

#### Main analysis

A mixed ANOVA was used to investigate food intake (grams of food consumed) with the following factors: food type (Vegetables and Non-Vegetables), delay (No Delay and Delay), message type (Neutral Control, Vegetable Variety Condition, Health, Descriptive Norm, and Liking Norm), and habitual vegetable consumption (low vs. high consumers) was determined by a median split (Robinson et al., 2014). To investigate effects for individual foods, the same model was used, but individual food type was included as a factor: cucumber, celery, broccoli, Pringles, tortillas, crackers, and dip. Significant interactions were investigated with follow-up ANOVA and Bonferroni-corrected *t*-tests, comparing message conditions to the Neutral Control condition.

#### Food liking

Individual food liking ratings were completed by participants for the foods that they consumed (they did not provide ratings for food items they did not eat). ANOVA was used to analyse these data (factors: message type and habitual vegetable consumption). Significant interactions and main effects were followed up as described above.

#### *Post-hoc* study data

Frequencies of responses derived from the open response data were analyzed with Pearson's chi-squared test (each message condition vs. Neutral Control).

## Results

### Food consumption^1^

#### Vegetables and non-vegetable consumption

There was a main effect of food type with participants eating a greater weight of vegetables than non-vegetables (114.9 vs. 58.8 g, respectively; *F*_(1, 333)_ = 177.02; *p* < 0.001; $\eta_{p}^{2}$ = 0.35) and a main effect of habitual vegetable consumption with low habitual consumers of vegetables eating less food overall compared to high consumers (79.2 vs. 94.5 g; *F*_(1, 333)_ = 7.94; *p* < 0.01; $\eta_{p}^{2}$ = 0.02). There was also a two-way interaction between food type and habitual consumption [*F*_(1, 333)_ = 7.82; *p* < 0.01; $\eta_{p}^{2}$ = 0.02], whereby low habitual consumers of vegetables ate a smaller weight of vegetables from the buffet than high consumers [99.3 vs. 127.3 g; *t*_(351)_ = −3.40; *p* < 0.01; $\eta_{p}^{2}$ = 0.03], however, there was no significant difference in consumption of non-vegetables by low and high consumers [57.2 vs. 59.6 g; *t*_(351)_ = −0.53; *p* > 0.05; $\eta_{p}^{2}$ = 0.00]. There were no main effects of or interaction with delay (all *p*s > 0.05). Main effects and interaction effects that are not mentioned were not significant (all *F*'s < 2.2, all *p*s > 0.05).

A marginal three-way interaction between food type, message, and habitual consumption was observed [*F*_(4, 333)_ = 2.28; *p* = 0.06; $\eta_{p}^{2}$ = 0.03]. Breaking down the interaction by food type, for vegetables there was a main effect of habitual vegetable consumption (as above—*p* < 0.01), and a significant two-way interaction between message and habitual consumption [*F*_(4, 333)_ = 2.44; *p* < 0.05; $\eta_{p}^{2}$ = 0.03]. Split by habitual consumption, there was no effect of message for the low consumers [Neutral Control = 82.1 g, Vegetable Variety condition = 125.8 g, Health = 101.3 g, Descriptive Norm = 89.9 g, and Liking Norm = 101.4 g; *F*_(4, 176)_ = 1.73; *p* = 0.1; $\eta_{p}^{2}$ = 0.04]. There was also no effect of message for the high consumers [Neutral Control = 143.7 g, Vegetable Variety condition = 114.6 g, Health = 130.1 g, Descriptive Norm = 136.7 g, and Liking Norm = 114.8 g; *F*_(4, 167)_ = 0.88; *p* = 0.5; $\eta_{p}^{2}$ = 0.02]. For non-vegetables there were no significant main effects or interactions (all *p*s > 0.05—refer to Table 1 for all means).

**Table 1:** **Consumption of vegetables and non-vegetables (in grams) split by message type and delay for low and high habitual consumers of vegetables (Standard error of the mean)**.

	**Neutral Control**		**Vegetable Variety Condition**		**Health Message**		**Descriptive Norm**		**Liking Norm**	
	**No delay**	**Delay**	**No delay**	**Delay**	**No delay**	**Delay**	**No delay**	**Delay**	**No delay**	**Delay**
**VEGETABLES**										
Low consumers	76.2 (15.5)	89.9 (17.8)	114.5 (17.4)	140.8 (20.1)	106.9 (17.4)	93.2 (20.8)	83.0 (18.8)	97.3 (19.4)	93.4 (15.9)	118.7 (23.4)
High consumers	157.8 (24.6)	132.9 (21.5)	127.0 (18.3)	103.0 (17.8)	131.7 (17.8)	128.6 (17.0)	139.3 (17.4)	134.3 (17.4)	115.4 (20.1)	114.3 (18.8)
**NON-VEGETABLES**										
Low consumers	64.8 (8.6)	44.4 (9.8)	64.7 (9.6)	66.6 (11.0)	41.7 (9.6)	48.4 (11.4)	61.4 (10.4)	64.4 (10.7)	57.2 (8.7)	57.3 (12.9)
High consumers	81.0 (13.5)	48.1 (11.9)	65.7 (10.1)	54.7 (9.8)	60.9 (9.8)	50.7 (9.3)	55.2 (9.6)	57.1 (9.6)	62.2 (11.1)	69.9 (10.4)

Hence, there was no effect of the liking norm on vegetable intake of low consumers overall. There was also no difference between the immediate and delay conditions.

#### Individual food item consumption

There was a main effect of food type [*F*_(4, 1230)_ = 155.60; *p* < 0.001; $\eta_{p}^{2}$ = 0.32], an effect of habitual vegetable consumption [*F*_(1, 331)_ = 8.23; *p* < 0.01; $\eta_{p}^{2}$ = 0.02], a two-way interaction between food type and habitual vegetable consumption [*F*_(6, 1986)_ = 4.59; *p* < 0.001; $\eta_{p}^{2}$ = 0.01] and a three-way interaction between food type, message, and habitual vegetable consumption [*F*_(24, 1986)_ = 1.53; *p* < 0.05; $\eta_{p}^{2}$ = 0.02], but no other main effects or interactions (all *p*s > 0.05). To investigate the three-way interaction, two-way ANOVAs (message and habitual vegetable consumption) were run on each individual food (see Table 2 for all data).

**Table 2:** **Consumption of buffet foods (in grams) split by message, delay, and low and high habitual consumption of vegetables (Standard error of the mean)**.

**Food Type**	**No delay**					**Delay**				
	**Neutral control**	**Vegetable variety condition**	**Health message**	**Descriptive norm**	**Liking norm**	**Neutral control**	**Vegetable variety condition**	**Health message**	**Descriptive norm**	**Liking norm**
**LOW HABITUAL CONSUMERS OF VEGETABLES**										
Participants	19	15	14	16	11	25	20	20	17	24
Cucumber	57.5 (10.6)	77.3 (11.9)	59.5 (11.9)	56.2 (12.9)	59.6 (10.8)	57.0 (12.2)	75.2 (13.7)	58.2 (14.2)	41.7 (13.3)	53.3 (16.0)
Celery	16.0 (7.1)	20.1 (7.9)	34.0 (7.9)	17.7 (8.6)	18.6 (7.2)	24.0 (8.1)	43.2 (9.2)	29.5 (9.5)	38.8 (8.9)	40.8 (10.7)
Broccoli	2.7 (4.1)	17.0 (4.6)	13.4 (4.6)	9.2 (5.0)	15.2 (4.2)	8.9 (4.7)	22.4 (5.3)	5.6 (5.5)	16.7 (5.1)	24.6 (6.2)
Pringles	22.2 (3.6)	20.0 (4.1)	10.0 (4.1)	26.7 (4.4)	20.2 (3.7)	16.8 (4.2)	17.8 (4.7)	17.1 (4.9)	21.4 (4.6)	20.2 (5.5)
Tortilla chips	18.4 (3.7)	23.5 (4.2)	16.1 (4.2)	20.9 (4.5)	17.8 (3.8)	15.7 (4.3)	22.9 (4.8)	21.4 (5.0)	25.4 (4.7)	20.4 (5.6)
Crackers	24.3 (4.1)	21.2 (4.6)	15.7 (4.6)	13.7 (4.9)	19.2 (4.2)	11.9 (4.7)	26.0 (5.3)	9.9 (5.5)	17.7 (5.1)	16.7 (6.2)
Dips	45.7 (9.9)	45.3 (11.1)	35.0 (11.1)	35.0 (12.0)	31.7 (10.1)	26.4 (11.4)	65.7 (12.8)	59.0 (13.3)	53.0 (12.4)	73.4 (15.0)
**HIGH HABITUAL CONSUMERS OF VEGETABLES**										
Participants	13	19	21	20	17	10	18	19	20	15
Cucumber	100.0 (16.8)	71.6 (12.5)	77.7 (12.2)	85.3 (11.9)	79.5 (13.7)	75.2 (15.3)	53.6 (12.5)	87.9 (11.6)	95.9 (11.9)	64.9 (12.9)
Celery	33.9 (11.2)	46.1 (8.4)	39.0 (8.1)	32.5 (7.9)	14.3 (9.2)	37.4 (10.2)	32.0 (8.4)	31.9 (7.7)	27.8 (7.9)	39.4 (8.6)
Broccoli	24.0 (6.5)	9.3 (4.8)	15.1 (4.7)	21.5 (4.6)	21.5 (5.3)	20.6 (5.9)	18.6 (4.8)	8.7 (4.5)	10.6 (4.6)	9.9 (5.0)
Pringles	17.9 (5.8)	19.7 (4.3)	21.8 (4.2)	17.1 (4.1)	21.8 (4.7)	13.4 (5.3)	14.1 (4.3)	19.2 (4.0)	15.6 (4.1)	19.8 (4.4)
Tortilla chips	34.1 (5.9)	24.2 (4.4)	19.1 (4.3)	20.2 (4.2)	20.4 (4.8)	19.9 (5.4)	21.0 (4.4)	14.8 (4.1)	22.9 (4.2)	25.9 (4.5)
Crackers	29.0 (6.5)	21.7 (4.8)	20.0 (4.7)	21.4 (4.6)	20.1 (5.3)	14.5 (5.9)	17.5 (4.8)	16.7 (4.5)	18.6 (4.6)	24.2 (4.9)
Dips	66.0 (15.7)	67.2 (11.7)	72.4 (11.4)	49.4 (11.1)	49.9 (12.8)	53.2 (14.3)	71.1 (11.7)	39.5 (10.8)	53.6 (11.1)	60.6 (12.0)

**Figure 2:**
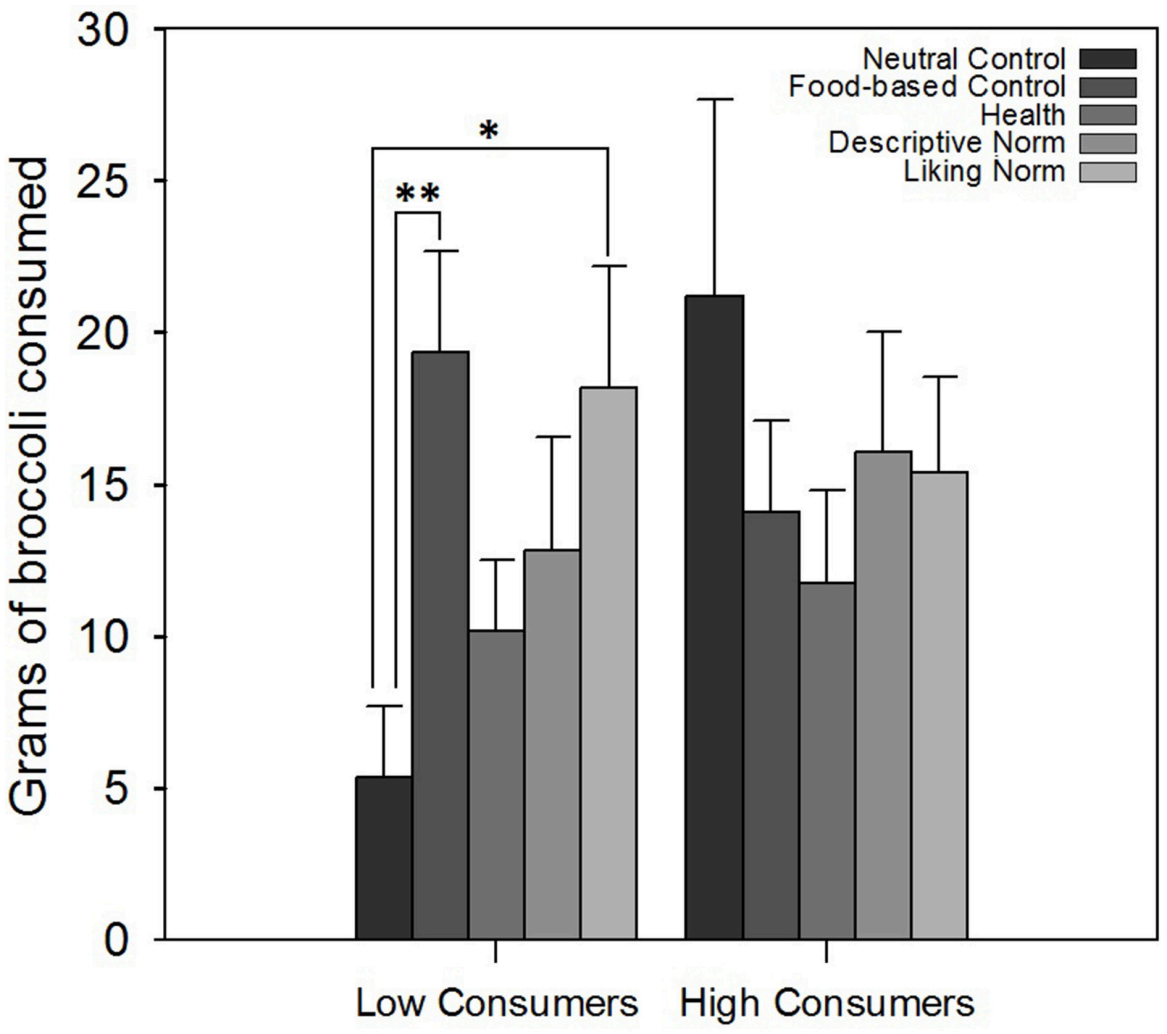
**Grams of broccoli consumed, split by message and habitual consumption of vegetables**. Participants in the Vegetable Variety and Liking Norm conditions ate significantly more broccoli than did those in the Neutral Control condition. Error bars indicate standard error of the mean. ^*^p < 0.05; ^**^p < 0.01.

For broccoli, there was an interaction between message and habitual consumption [*F*_(4, 343)_ = 2.50; *p* < 0.05; $\eta_{p}^{2}$ = 0.03], but no main effect of message or of habitual vegetable consumption (both *p*s > 0.05). Low consumers ate more broccoli in the Liking Norm condition [*t*_(77)_ = 2.77; *p* < 0.05; $\eta_{p}^{2}$ = 0.10] and in the Vegetable Variety condition [*t*_(77)_ = 3.41; *p* < 0.01; $\eta_{p}^{2}$ = 0.14] than in the Neutral Control condition (see Figure 2). There were no differences in intake among the high habitual vegetable consumers (all *p*s > 0.05).

For cucumber there was an effect of habitual vegetable consumption [*F*_(1, 343)_ = 11.29; *p* < 0.001; $\eta_{p}^{2}$ = 0.03], and an interaction between message type and habitual vegetable consumption [*F*_(4, 343)_ = 2.99; *p* < 0.05; $\eta_{p}^{2}$ = 0.03], but no main effect of message [*F*_(4, 343)_ = 0.21; *p* > 0.05; $\eta_{p}^{2}$ = 0.002]. Breaking down the interaction, Bonferroni-corrected *t*-tests revealed that there was no significant difference in consumption between the Neutral Control message and the other messages, for either the low or the high consumers (all *p*s > 0.05).

For the dip, there was a main effect of habitual vegetable consumption whereby high vegetable consumers ate more dip than did low vegetable consumers (58.1 vs. 45.0 g vs.; *F*_(1, 341)_ = 5.82; *p* < 0.05; $\eta_{p}^{2}$ = 0.02), but no effect of message, nor interaction between message and habitual vegetable consumption (both *p*s > 0.05). For the remaining foods (celery, Pringles, tortillas, and crackers) there were no significant main effects or interactions (all *p*s > 0.05).

### Manipulation check

When asked what they thought the aims of the study were with an open ended response question, 3.1% of participants correctly guessed the study aims. When asked whether they thought the “poster study” might have affected their behavior in the “appetite and mood study,” 31.4% reported that they thought it might have affected their eating behavior. Finally, when told the precise aims of the study and asked to indicate with a yes or no response whether they had become aware of these aims during the study, 22.7% of the sample claimed that they had become aware.

### Participant characteristics and baseline measures

Participant characteristics and baseline measures were analyzed as a randomization check. For the following measures there was no significant difference between the conditions: BMI, TFEQ scores (cognitive restraint, disinhibition, and hunger), and VAS scores (appetite, negative mood, negative physical effects, alertness, and drowsiness; pre-meal ratings only) (all *p*s > 0.05, see Table 3 for breakdown by message type). However, for age there was a main effect of message type [*F*_(4, 348)_ = 5.65; *p* < 0.001; $\eta_{p}^{2}$ = 0.06], whereby age was significantly lower in the Health message, Descriptive Norm, and Liking Norm message conditions, compared with the neutral control message (all *p*s < 0.01, see Table 3). Pearson's correlation revealed that age was not associated with food intake (all *p*s > 0.05) and so age was not controlled for in the analysis of food intake.

**Table 3:** **Participant characteristics and baseline measures split by message type (Standard error of the mean)**.

**Measure**	**Neutral control**	**Vegetable variety condition**	**Health message**	**Descriptive norm**	**Liking norm**
Number of participants	67	72	74	73	67
Age	23.3 (0.6)	21.6 (0.5)	21.1 (0.3)^**^	21.0 (0.4)^**^	20.7 (0.3)^***^
Body mass index (BMI)	23.1 (0.5)	22.1 (0.4)	23.1 (0.4)	22.8 (0.4)	22.2 (0.3)
TFEQ cognitive restraint (range = 0–21)	8.0 (0.6)	8.6 (0.6)	9.5 (0.6)	8.3 (0.5)	9.1 (0.7)
TFEQ disinhibition (range = 0–16)	7.3 (0.4)	7.0 (0.4)	7.6 (0.4)	7.7 (0.4)	7.1 (0.4)
TFEQ hunger (range = 0–14)	7.3 (0.4)	6.5 (0.4)	7.0 (0.4)	7.0 (0.5)	6.7 (0.4)
Habitual consumption of vegetables	2.1 (0.2)	2.8 (0.2)	2.5 (0.1)	2.6 (0.2)	2.3 (0.1)
Servings of vegetables a typical student is believed to eat each day	2.1 (0.1)	1.9 (0.1)	2.1 (0.1)	1.8 (0.1)	1.7 (0.1)^*^
**VISUAL ANALOG SCALES**					
Appetite	66.0 (1.8)	63.0 (1.7)	68.0 (1.9)	64.6 (1.6)	64.6 (2.3)
Negative mood	20.2 (1.3)	19.8 (1.4)	21.7 (1.7)	23.5 (1.5)	19.7 (1.5)
Negative physical effects	15.6 (1.6)	12.8 (1.4)	12.7 (1.4)	16.6 (1.9)	12.6 (1.8)
Alertness	60.7 (3.4)	66.5 (2.5)	57.4 (2.9)	62.0 (2.2)	64.3 (3.2)
Drowsiness	23.8 (2.6)	24.9 (2.3)	34.3 (3.1)	28.3 (2.5)	25.6 (27.5)

* *p < 0.05*,

** *p < 0.01*,

*** *p < 0.001*.

Baseline perceptions of the norm were assessed (“Servings of vegetables a typical student is believed to eat each day”) showing a main effect of message type [*F*_(4, 345)_ = 2.72; *p* < 0.05; $\eta_{p}^{2}$ = 0.03], whereby participants in the Liking Norm condition believed that students ate fewer vegetables daily, compared to the Neutral Control condition [*F*_(1, 130)_ = 7.56; *p* < 0.05; $\eta_{p}^{2}$ = 0.06, see Table 3 for means]. Perceptions of the norm were not related to food intake (all *p*s > 0.05) so this was not controlled for in the analysis of food intake. The two measures of a participant's own vegetable intake (taken toward the end of the study) were significantly positively correlated (*r* = 0.75, *p* < 0.001), hence, they were averaged to form a single measure of habitual consumption of vegetables (Table 3). There were no significant differences between message types for this measure [*F*_(4, 348)_ = 2.30; *p* > 0.05; $\eta_{p}^{2}$ = 0.03].

### Poster ratings

One-way ANOVA showed no difference between the conditions for ratings of either poster understanding or professional appearance (both *p*s > 0.05). However, for poster legitimacy, there was a main effect of message type [*F*_(4, 348)_ = 3.28; *p* < 0.05; $\eta_{p}^{2}$ = 0.04] whereby poster legitimacy was rated significantly higher in the Health Message condition than in all the other conditions (*p*s < 0.05, see Table 4). This measure was not significantly associated with intake (all *p*s > 0.05).

**Table 4:** **Poster ratings split by message type (Standard error of the mean)**.

**Measure**	**Neutral control**	**Vegetable variety condition**	**Health message**	**Descriptive norm**	**Liking norm**
Poster understanding (range = 1–5)	4.4 (0.1)	4.4 (0.1)	4.5 (0.1)	4.4 (0.1)	4.3 (0.1)
Professional appearance (range = 1–5)	3.0 (0.1)	2.9 (0.1)	2.9 (0.1)	2.9 (0.1)	2.7 (0.1)
Poster legitimacy (range = 1–5)	3.5 (0.1)	3.5 (0.1)	3.8 (0.1)^*^	3.4 (0.1)	3.5 (0.1)

* *p < 0.05*.

### Food liking

For cucumber, there was a main effect of habitual consumption [*F*_(1, 298)_ = 8.82; *p* < 0.01; $\eta_{p}^{2}$ = 0.03) whereby low habitual consumers of vegetables rated the cucumber as less liked than did the high consumers (74.7 vs. 82.4 mm—see Table 5). There was also a significant two-way interaction between habitual consumption and message type [*F*_(4, 298)_ = 2.82; *p* < 0.05; $\eta_{p}^{2}$ = 0.04], but no main effect of message type (*p* > 0.05). Examination of the main effect of message type in low and high consumers separately showed a trend for an effect in low (but not high) consumers [*F*_(4, 148)_ = 2.214; *p* = 0.07; $\eta_{p}^{2}$ = 0.06], whereby Bonferroni-corrected *t*-tests showed that cucumber liking was significantly higher in the vegetable variety condition compared to the neutral control condition (86.8 vs. 72.0 mm, respectively; *p* < 0.05).

**Table 5:** **Liking ratings of each food split by message type for low and high habitual consumers of vegetables (Standard error of the mean)**.

**Participants**	**Neutral control**	**Vegetable variety condition**	**Health message**	**Descriptive norm**	**Liking norm**
**LOW HABITUAL CONSUMERS OF VEGETABLES**					
Cucumber	72.0 (3.7)	86.8 (3.9)	73.1 (4.2)	69.1 (4.5)	72.5 (4.2)
Celery	48.6 (5.3)	63.0 (6.5)	54.7 (6.1)	42.1 (6.1)	55.8 (6.1)
Broccoli	34.9 (8.2)	44.2 (6.1)	25.0 (7.1)	33.3 (7.3)	39.2 (5.8)
Pringles	80.7 (4.3)	68.9 (4.6)	76.5 (4.7)	74.6 (4.6)	75.3 (4.6)
Tortilla chips	70.1 (3.5)	77.4 (3.8)	82.1 (3.9)	78.3 (3.9)	80.6 (3.8)
Crackers	68.6 (4.4)	71.7 (4.8)	68.7 (5.3)	66.6 (5.1)	68.9 (5.2)
**HIGH HABITUAL CONSUMERS OF VEGETABLES**					
Cucumber	80.1 (5.0)	78.8 (4.0)	88.0 (3.7)	83.9 (3.5)	81.4 (4.3)
Celery	49.7 (7.0)	68.8 (5.6)	63.4 (5.8)	60.9 (5.8)	66.3 (7.2)
Broccoli	52.0 (8.8)	56.3 (6.6)	39.7 (6.6)	46.5 (6.8)	37.4 (6.2)
Pringles	60.0 (5.7)	65.7 (4.5)	75.3 (4.5)	69.8 (4.3)	64.1 (4.8)
Tortilla chips	74.8 (4.7)	76.0 (3.9)	81.4 (3.8)	82.7 (3.7)	72.2 (4.0)
Crackers	77.8 (6.0)	68.7 (4.7)	72.6 (5.1)	69.7 (4.6)	70.6 (5.1)

For celery, there was a main effect of habitual consumption [*F*_(1, 253)_ = 5.29; *p* < 0.05; $\eta_{p}^{2}$ = 0.02], whereby low habitual consumers of vegetables rated the celery as less liked than did the high consumers (52.8 vs. 61.8 mm). There was also a main effect of message type [*F*_(4, 253)_ = 2.54; *p* < 0.05; $\eta_{p}^{2}$ = 0.04], whereby Bonferroni-corrected *t*-tests showed that liking of celery was significantly higher in the vegetable variety condition compared to the neutral control condition (49.0 vs. 66.4 mm, respectively; *p* < 0.05); there was also a similar trend for enhanced ratings in the liking norm condition compared to the neutral control condition (49.0 vs. 60.2 mm, respectively; *p* = 0.08). There was no significant interaction between message type and habitual consumption *p* > 0.05).

For broccoli, there was a main effect of habitual consumption [*F*_(1, 208)_ = 6.29; *p* < 0.05; $\eta_{p}^{2}$ = 0.03], whereby low habitual consumers of vegetables rated the broccoli as less liked than did the high consumers (35.3 vs. 46.4 mm). There was no main effect of message or significant interaction (both *p*s > 0.05). A similar pattern was also noted for pringles; there was a main effect of habitual consumption [*F*_(1, 296)_ = 7.66; *p* < 0.01; $\eta_{p}^{2}$ = 0.03], whereby low habitual consumers of vegetables rated the pringles as more liked than did the high consumers (75.2 vs. 67.0 mm), but no main effect of message or significant interaction (both *p*s > 0.05). For tortillas and crackers there were no main effects or significant interaction (all *p*s > 0.05).

### *Post-hoc* poster study

As expected, compared to the Neutral Control condition, more participants: noted references to health in the Health message condition; noted references to norms in the Descriptive Norm message condition; and noted references to norms and vegetable liking in the Liking Norm condition (all *p*s < 0.001—see Table 6). Participants thought that the Vegetable Variety condition was more likely than the Neutral Control message to increase the variety of new vegetables consumed (*p* < 0.001) and participants in the Vegetable Variety condition referenced vegetable liking more so than those in the Neutral Control condition (*p* < 0.05). Also, participants thought that each poster (Vegetable Variety condition, Health, Descriptive Norm and Liking Norm) was more likely to increase the amount of vegetables consumed compared to the Neutral Control poster (all *p*s < 0.05).

**Table 6:** **Frequencies of responses under the five key themes, split by message**.

**Theme**	**Neutral control**	**Vegetable variety condition**	**Health message**	**Descriptive norm**	**Liking norm**
Reference to health	0	2	21^***^	3	4
Reference to norms	0	0	0	17^***^	14^***^
Reference to vegetable liking	0	7^*^	0	0	16^***^
Increase variety of new vegetables consumed	0	32^***^	0	1	4
Increase amount of vegetables consumed	0	8^*^	34^***^	26^***^	19^***^

* p < 0.05;

*** *p < 0.001*.

One-way ANOVA showed no effect of message condition for “Portions of vegetables you eat per day” and “Recommended portions of vegetables you should eat per day,” but there was a main effect of message condition for all remaining items (all *p*s < 0.01—see Table 7). Bonferroni-corrected *t*-tests showed that participants thought that all posters (compared to the Neutral Control) would: increase the consumption of vegetables eaten by other people (all *p*s < 0.01) and would enhance the liking of vegetables by other people (all *p*s < 0.01). For the Vegetable Variety condition, Descriptive Norm and Liking Norm (but not Health), participants believed that a higher percentage of the population were consuming the recommended portion of vegetables (all *p*s < 0.05). Participants also thought that people would eat more portions of vegetables after reading all of the posters, compared to the Neutral Control (all *p*s < 0.05).

**Table 7:** **Ratings of posters split by message (Standard error of the mean)**.

**Measure**	**Neutral control**	**Vegetable variety condition**	**Health message**	**Descriptive norm**	**Liking norm**
Portions of vegetables you eat per day	2.8	2.9	2.7	2.7	2.8
	(0.2)	(0.3)	−0.2	(0.3)	(0.3)
Will poster influence amount of vegetables eaten by other people?	46.1	63.4^***^	72.6*** (3.4)	67.1*** (2.9)	62.5^**^
	(2.0)	(2.0)	(3.4)	(2.9)	(2.7)
Will poster influence liking of vegetables by other people?	44.8	56.9^**^	60.2^**^	61.3*** (2.8)	62.1^**^
	(2.0)	(92.6)	(3.1)	(2.8)	(3.5)
Recommended portions of vegetables you should eat per day	4.1	4.3	4.2	4.1	4.2
	(0.2)	(0.2)	(0.2)	(0.2)	(0.2)
Proportion of the population consuming recommended portions of vegetables	39.9	44.9^*^	43.9	48.7^*^	50.0^*^
	(3.8)	(3.6)	(3.6)	(3.7)	(3.8)
Portions of vegetables people eat per day based on the poster you have read	2.2	2.6^*^	2.8^*^	3.3^***^	3.3^***^
	(0.2)	(0.2)	(0.2)	(0.2)	(0.2)

* p < 0.05;

** p < 0.01;

*** *p < 0.001*.

## Discussion

The liking norm did not significantly enhance the intake of all vegetables in low habitual consumers of vegetables. However, it appeared to enhance the consumption of raw broccoli by this population. This effect did not significantly differ between those participants who were given immediate access or delayed access to a buffet, suggesting the effect persists beyond initial exposure for at least 24 h. A message suggesting that there are lots of different types of vegetables in the world (vegetable variety condition) also significantly increased the consumption of raw broccoli. Unlike previous research we did not observe a significant effect of the descriptive norm on vegetable intake. We did not observe any effect of the health message either; however, this was not necessarily surprising given the limited effects associated with health-based interventions to improve healthy eating (Rekhy and McConchie, 2014).

The ability of the liking norm message to increase broccoli intake to more than three times the amount that was consumed in the control condition suggests that referring to the enjoyment of vegetables may be a useful strategy in interventions aimed at promoting vegetable intake. We expected that the liking norm would increase the intake of all the vegetables and that it might be especially effective in increasing intake of a less liked vegetable. Indeed, broccoli was the least-liked vegetable (based on the available liking data provided after the buffet) and so it is possible that the low baseline liking contributed to the effect of the liking norm. Although, it would be useful to clarify the nature of this selective effect, the increase in consumption of a cruciferous vegetable is of interest as it is particularly difficult to increase intake of more bitter tasting vegetables (Johnston et al., 2000). It should be noted, however, that the results are based on comparisons of conditions with relatively small numbers of participants and therefore caution is warranted interpreting these results.

To our knowledge, this study provides the first evidence that social norm effects on food intake persist beyond initial exposure for a full day. It should be noted that this interpretation is based on the null interactions with the delay factor in the ANOVA. However, inspection of the means shows that broccoli consumption by low consumers in the delayed condition was higher than in the no-delay condition for both the liking norm and vegetable variety conditions (albeit non-significantly) which is consistent with the idea that the norm effects do not decay over time. The mechanism underlying this effect is unclear; however, one possibility is that these messages lead to a change in the hedonic evaluation of vegetables (i.e., other students really do like vegetables so I must like them too) which facilitates a stable enhancement of vegetable consumption. Although, the liking ratings for broccoli were not significantly affected by either the liking norm or vegetable variety condition, the latter significantly enhanced the liking of cucumber in low habitual consumers of vegetables. For both low and high habitual consumers, the vegetable variety condition increased liking for celery and there was a trend for a similar effect of the liking norm too. More generally, there was a pattern for higher liking ratings of all three vegetables for low consumers exposed to vegetable variety and liking norm conditions, similar to the pattern seen with the consumption data. These data suggest that enhanced liking may contribute to changes in intake. However, further work is necessary to confirm the hypothesis that liking is a mechanism by which social norms exert their effects on consumption, especially because of the low power of the present analysis due to the fact that not everyone tasted all the vegetables. In the longer term (>24 h) there are additional considerations. For instance, would subsequent exposure to the same social norm information continue to maintain the effect, or would participants eventually habituate to the information and the effect diminish? Alternatively, might cumulative exposure enhance the effect, further boosting the consumption of vegetables? Further, work to answer these questions would help to determine whether social norm-based interventions can promote long-term changes in food choice.

The effect of the vegetable variety condition on broccoli consumption was unexpected, but to some extent clarified by the *post-hoc* study on the poster messages. Participants thought that the message would increase the amount and variety of new vegetables that people consume and how much people like vegetables. Participants also thought a greater percentage of the population was consuming the recommended amount after viewing this poster (similar to the liking and descriptive norm poster), suggesting that it was also influencing the perception of vegetable consumption norms. Together, these results suggest that the poster was affecting perceptions of liking, intake (both amount and variety), and norms, which might explain why it had the greatest influence on broccoli consumption. Given that subtle health messages on food choice are more successful than are explicit health messages (Wagner et al., 2015) and that people are less likely to demonstrate reactance to messages that are more suggestive than restrictive (Stok et al., 2015), it is possible that our message—stating a fact, without any suggestion as to how to behave—meets the criteria for an effective message to change behavior. It might be advantageous to explore this type of message, because unlike the normative messages, this type of message does not require any data on the behavior of a group (which can be difficult to acquire). In the context of the selective effect on broccoli, it is plausible that of the vegetables in the buffet, participants would have been least likely to have consumed raw broccoli outside of the laboratory. Therefore, consuming it from the buffet would be the simplest way to increase one's vegetable variety during the test session, if as the *post-hoc* study suggested, people felt more motivated to increase their variety of vegetables.

We expected to see an effect of the descriptive norm in this study. However, most of the laboratory work examining food intake and descriptive norms has focussed on fruit, or fruit, and vegetables (e.g., Robinson et al., 2014; Stok et al., 2014). Relatively few studies have examined the effect of descriptive norms on a vegetable-only buffet, much less one consisting of exclusively green (bitter) vegetables; others have used less bitter vegetables such as carrots, tomatoes, sweet-corn, etc. Hence, the success of descriptive norms might be linked to the sweetness or palatability of these foods (or indeed, other characteristics of these foods). Another possibility is that the low consumers in this study were not actually that low. In our study mean vegetable servings per day for the low consumers were 1.4 portions, whereas others have reported consumption in the region of 0.7 portions for their low consumers (Robinson et al., 2014). Hence, it might be the case that the low consumers in this study had a reduced capacity to show an increase in intake, limiting the ability to detect an effect of the descriptive norm. The way in which the posters were worded to convey a majority (descriptive norm, “most students;” liking norm, “80% of students”) may also have contributed to detecting a liking norm but not a descriptive norm effect. However, the results of the poster evaluation study suggest that people think the posters would be equally effective in increasing vegetable consumption. It should also be noted that those in the descriptive norm condition consumed more than twice the amount of broccoli than those in the neutral control condition. The direction and size of this effect is in line with previous work (Robinson et al., 2014), hence, it may also be the case that power to detect an effect of the descriptive norm was an issue.

Reassuringly, there were no significant effects on the consumption of the energy-dense food items. This suggests that enhanced intake of a healthy option does not produce compensatory effects, or licensing of a less healthy option, which is critical for a successful healthy eating intervention. However, it is not known whether individuals engaged in any compensatory behaviors after finishing the study, so further work to examine this possibility is required.

In conclusion, we show that in a laboratory setting both a liking norm and a vegetable variety condition increased the consumption of a cruciferous vegetable among habitual low vegetable consumers. This effect was still evident after 24 h, although we found little evidence that these messages increased consumption of other vegetables. These findings are suggestive that social norm based messages about others' liking may be effective at increasing consumption of lesser liked vegetables, although future work is needed confirm this and the mechanism by which this message works.

## Author contributions

JT, ER, PA, CH and SH contributed to the design of the research. JT and JL collected the study data and JT and SH analyzed the data together. All authors critically reviewed the manuscript and improved it. All authors had access to all the data and take responsibility for the integrity of the data and the accuracy of the data analysis.

## Funding

The study was funded by a grant from the Economic and Social Research Council (ESRC—ES/K002678/1). JT is funded by an ESRC Grant (ES/K002678/1) of which PA and CP are co-investigators and SH is the principal investigator. ER is part of a research team that has received research funding from Unilever and the American Beverage Association.

### Conflict of interest statement

The authors declare that the research was conducted in the absence of any commercial or financial relationships that could be construed as a potential conflict of interest. The reviewer KV and handling Editor declared a current collaboration and the handling Editor states that the process nevertheless met the standards of a fair and objective review.

## Footnotes

^1^Inclusion of data from individuals who did not correctly recall the poster messages did not change the overall pattern of results. However, for the consumption of broccoli; the *p*-value for the interaction between message and habitual vegetable consumption for the low consumers increased to 0.054 and the subsequent *p-*value for the comparison between the neutral control and the liking condition increased to 0.055.
